# On Solute Recovery and Productivity in Chiral Resolution through Solid-State Deracemization by Temperature Cycling

**DOI:** 10.1021/acs.cgd.4c00233

**Published:** 2024-04-23

**Authors:** Mercedeh Sadat Hosseinalipour, Leif-Thore Deck, Marco Mazzotti

**Affiliations:** Institute of Energy and Process Engineering, ETH Zurich, 8092 Zurich, Switzerland

## Abstract

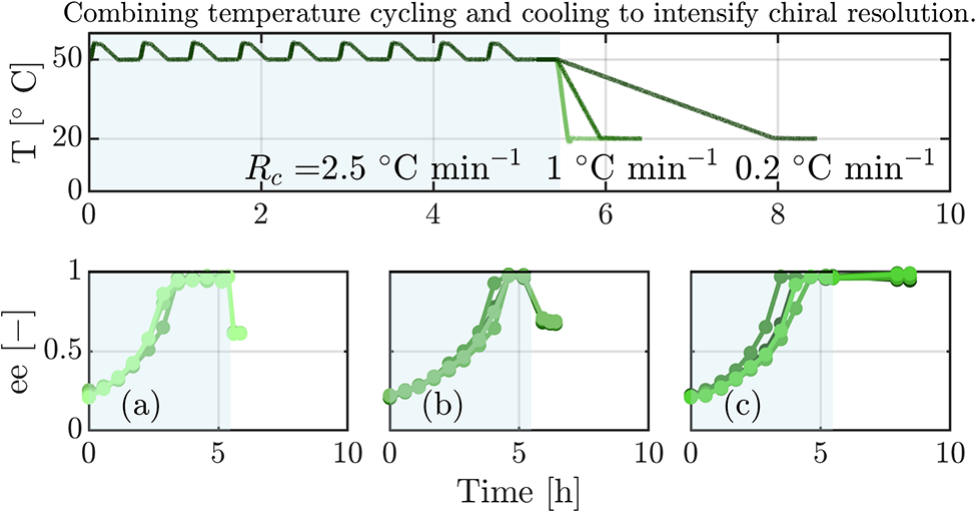

Temperature cycling represents an effective means for the deracemization of chiral compounds that crystallize as conglomerates and racemize in solution. In such a process, a suspension enriched in the desired enantiomer is converted into an enantiopure one through periodic cycles of crystal dissolution and crystal growth. We show that performing temperature cycling at higher temperatures leads to faster deracemization and, consequently, higher productivity. However, this comes at the cost of lower recovery, as the solution contains potentially relevant amounts of solute due to the higher solubility at an elevated temperature. In this work, we introduce and compare two process variants that mitigate this issue. The first involves temperature cycling, followed by linear cooling, whereas the second is based on merging the temperature cycles and cooling crystallization. Experiments carried out with the chiral compound *N*-(2-methylbenzylidene)-phenylglycine amide show that the former variant is faster than the latter, and it is easier to design and implement. In this process, the choice of an appropriate cooling rate is essential to avoid nucleation of the undesired enantiomer.

## Abstract 2

This contribution on solid-state deracemization explores strategies to improve solute recovery and productivity. Carrying out temperature cycles at elevated temperatures leads to high productivity but also results in low solute recovery. To address this trade-off, two process extensions were investigated, and recommendations for their implementation are provided.

## 1 Introduction

Chiral compounds exist as pairs of enantiomers that are mirror images of one another and exhibit identical physical and chemical properties in nonchiral (achiral) environments. Yet, their behavior differs significantly within chiral environments. This is particularly relevant in biological systems, which are predominantly homochiral, meaning that of the chiral species existent only one out of the two enantiomeric forms is present; this is observed for instance in both amino acids and sugars.^1−7^ Chiral molecules can form three distinct structures upon crystallization: (I) a chiral conglomerate, where the enantiomers crystallize separately into enantiopure L and D crystals; (II) a racemic DL crystal that incorporates both enantiomers in a 1:1 ratio within a regular crystal lattice; and (III) a solid solution, where enantiomers are incorporated into a single crystal in a nonstoichiometric proportion.^8,9^ In the context of chemical synthesis, the synthesis of a chiral molecule from an achiral feedstock generally yields a racemate. In contrast, given the importance of chirality on the biological efficacy of, for instance, active pharmaceutical ingredients,^10,11^ regulatory bodies increasingly mandate the isolation of the desired enantiomer (termed eutomer).^12,13^ This necessitates the development of effective separation methods for enantiomers.^14^ In particular, techniques based on crystallization have attracted interest in the separation of conglomerate-forming compounds, as they promise high purity at comparably low costs.^15−17^ These techniques can be divided into two groups.

First, there are processes that focus on the recovery of the solute from the solution via seeded crystallization of the pure desired enantiomer, such as preferential crystallization,^18−20^ where molecules of the desired enantiomer grow onto the seed crystals. The yield of such processes is limited by the absence of enantiomeric conversion in the solution; hence, its maximum is 50%. If, however, a racemization reaction in the liquid phase takes place, for example through addition of a catalyst, a yield of 100% can be attained.^21−24^

Then, there is a second group of techniques that targets the deracemization of an initial suspension enriched in the crystals of one enantiomer via various technical means, such as temperature cycling,^25−31^ solvent cycling,^32,33^ abrasive grinding,^34−38^ or high-pressure homogenization.^39^ In recent years, there has been extensive research on deracemization via temperature cycling due to its ease of implementation and control. This process achieves complete deracemization through repetitive cycles of crystal growth at a low temperature and crystal dissolution at a high temperature. The intricate interplay of crystal growth, dissolution, and racemization reaction significantly influences the final purity and productivity of the process.^40,41^ The governing mechanism behind deracemization via temperature cycling and related processes has been a topic of interest in the scientific literature over the past two decades.^31,42,43^ Using theoretical analysis and numerical simulations, we have shown recently^44^ that deracemization happens if, and only if, under rather general conditions dissolution is faster than growth; neither crystal agglomeration, nor breakage, nor ripening are required, all of which have been considered essential in earlier process models, largely because the first reports of deracemization have been on the technical variations based on grinding.^34,45^

This study builds on our earlier theoretical and experimental work^27,40,44^ and aims at establishing conditions that enable deracemization with high productivity and high solute recovery. Experimental findings indicate that the deracemization of *N*-(2-methylbenzylidene)-phenylglycine amide (NMPA) occurs more rapidly at higher temperatures,^26^ resulting in increased productivity. Once enantiopure crystals are obtained at the end of the process, the remaining solution, which still contains a relevant amount of solute due to its higher solubility at higher temperatures, can either be discarded or recycled for use in a subsequent process. This highlights that when choosing optimal conditions there is a trade-off between process productivity and solute recovery. We aim to address this trade-off as follows. First, we experimentally compare the deracemization rate of NMPA at low (20 °C) and high (50 °C) temperature levels. We then explore two process variants that both enhance the recovery of the solute but come with different constraints in terms of productivity and enantiopurity of the product. The first variant involves temperature cycling followed by a linear cooling step, termed “TC + C”, whereas the second is based on the integration of temperature cycles into cooling crystallization, termed deracemization via cooling temperature cycling, “CTC”. The experimental results are presented in Section 3, the experimental methods are discussed in Section 4 and the relevant conclusions are summarized in Section 5.

## 2 Trade-off between Recovery and Productivity

Here we experimentally study the deracemization of NMPA at elevated temperatures (50 °C, E_1_) and at ambient temperature (20 °C, E_2_). To design and compare different process variants, we consider five parameters, following common practice in the literature.^20,22,27^ First, the enantiomeric excess (ee) of the crystalline phase, which quantifies the enantiopurity of the suspension
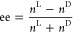
1where *n*^L^ represents the suspension density (in g g_s_^–1^, defined as the mass of suspended crystals per unit mass of solvent) of the majority (L) enantiomer and *n*^D^ represents that of the minority (D) enantiomer. Second, productivity quantifies the amount of desired crystals produced per unit of time and per unit mass of solvent. Third, recovery denotes the fraction of solute material that ends up in the final product, here, the crystalline form, with respect to the total amount of solute used in the process. Ideally, this quantity attains a value of one; however, since some solute remains in the solution at the end of the process, values smaller than one are observed. Fourth, the dissolution factor δ characterizes the operating conditions of a deracemization process in a physically intuitive manner, as its inverse represents the fraction of the minority enantiomer crystals that dissolve during the heating step of a sufficiently long temperature cycle. The parameter δ_0_ is the dissolution factor for the first cycle^27^
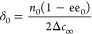
2where ee_0_ is the enantiomeric excess in suspension at the beginning of the experiment, *n*_0_ is the total initial suspension density in g g_s_^–1^, and Δ*c*_∞_ in g g_s_^–1^ is the difference in solubility of either of the enantiomers between the maximum, *T*_max_, and the minimum, *T*_min_, temperature in a cycle. The subscript ‘s’ refers to the solvent throughout this work. Fifth and last, the cycle efficiency η denotes how much material is transformed during a single cycle from the minor enantiomer to the major enantiomer, defined as a fraction of the solubility difference Δ*c*_∞_. For example, a value of one, which represents the theoretical maximum, would indicate that the amount of material that is transformed during one cycle equals the value of Δ*c*_∞_. An exact expression for η in a temperature-cycling process has been derived previously.^44^ Further, an estimate of η can be computed from experimental data as
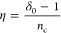
3where *n*_c_ is the experimentally observed number of cycles required for complete deracemization.

**Figure 1:**
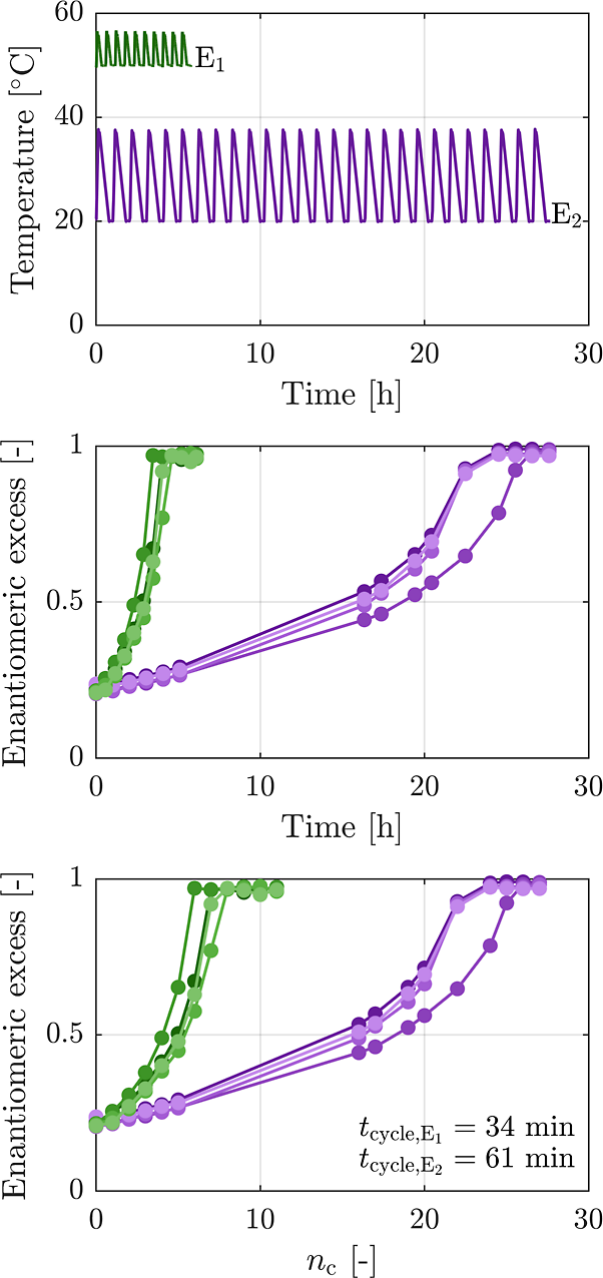
Experimental evolution of ee for the deracemization of suspensions with *n*_0_ = 0.04 g g^–1^, ee_0_ = 0.2, and δ_0_ = 2.4 for *T*_min_ = 50 °C in E_1_ and *T*_min_ = 20 °C in E_2_. The ee is measured at the end of a cycle’s low temperature step. E_1_ reached high purity in 9 cycles and E_2_ in 25 cycles. The calculated cycle efficiencies for these two conditions are η_E_1__ = 0.15 and η_E_2__ = 0.06, respectively.

A temperature cycling process comprises several temperature cycles, each of which consists of four steps: a heating ramp with heating rate *R*_h_, an isothermal step at high temperature, a cooling ramp with cooling rate *R*_c_, and an isothermal step at low temperature. Experiments were carried out with an initial suspension density of *n*_0_ = 0.04 g g_s_^–1^ unless otherwise specified. Further, two values of the lower temperature level *T*_min_ were investigated, namely 50 °C and 20 °C, whereas *T*_max_ has been chosen such that the resulting dissolution factors for the two cases are the same, namely δ_0_ = 2.4. Figure 1 shows the evolution of ee during these experiments, whereby the lines identify independent replicates and the colors of the different cycles, particularly the different temperature levels. Further information about the experimental protocol is reported in Sections 4.1 and 4.2.

The main conclusions that can be drawn from the data shown in Figure 1 are that (i) complete deracemization is achieved for both temperature cycle experiments, i.e., at both temperature levels, in a reproducible manner (see Section 4.3 for a more in-depth discussion on reproducibility); and (ii) deracemization is significantly faster at 50 °C compared to 20 °C, as the experiments reached enantiopurity in 9 and in 25 cycles, respectively. This is despite the fact that the individual cycles are longer at 20 °C due to longer heating and cooling ramps. Given the dissolution factor of δ_0_ = 2.4, these observations result in cycle efficiencies of 0.15 at 50 °C and 0.06 at 20 °C. These values are similar to those computed earlier for deracemization experiments of NMPA under rather different operating conditions, where we observed efficiency values in the range from 0.05 to 0.20.^44^

It may appear physically intuitive that deracemization proceeds faster at higher temperatures, as the kinetics of crystal growth, dissolution, and racemization reaction all accelerate with increasing temperature. Providing an exact explanation for this behavior, however, is far from trivial. Based on the theoretical analysis reported earlier,^44^ we formulate two arguments for why deracemization accelerates with temperature.

First, we acknowledge that deracemization is driven by changes in solubility, which are induced by changes in the temperature. The suspension reacts to such changes through crystal growth or dissolution, as the concentration of the solute in solution evolves toward the new solubility level, i.e., toward equilibrium at the new temperature level. The time required to do so depends on the kinetics of growth and dissolution, which both accelerate with the temperature. Hence, at higher temperatures, the suspension reacts more quickly to the solubility change, which allows shortening the duration of the individual cycles without penalizing the cycle efficiency. This argument holds independent of how fast crystallization kinetics indeed are, thus implying that higher temperatures are generally beneficial for the productivity of deracemization.

Second, it has been shown previously that deracemization accelerates when the chemical reaction is faster, which is the case at higher temperatures.^26,40^ We could rationalize this within our earlier analysis, which revealed that the ratio between the characteristic time of growth/dissolution, on the one hand, and that of the reaction, on the other hand, strongly affects the value of the cycle efficiency. Higher cycle efficiencies are observed with increasing temperature when the temperature dependence of the reaction rate is stronger than that of the growth/dissolution rate. Given that the precise kinetics of crystal growth/dissolution for NMPA has not yet been measured, it is not possible to conclude whether this argument applies to the experiments reported in this work or not.

In the following, we assume that the experimental trends observed here, namely that deracemization accelerates with temperature, exhibit some generality, and therefore we investigate how to design deracemization processes with both high productivity and high solute recovery. In particular, we emphasize that operating at high temperatures may be associated with a relevant amount of solute in solution due to the higher solubility level. Therefore, recovering the solute becomes an attractive proposition at the end of the temperature cycling process.

**Figure 2:**
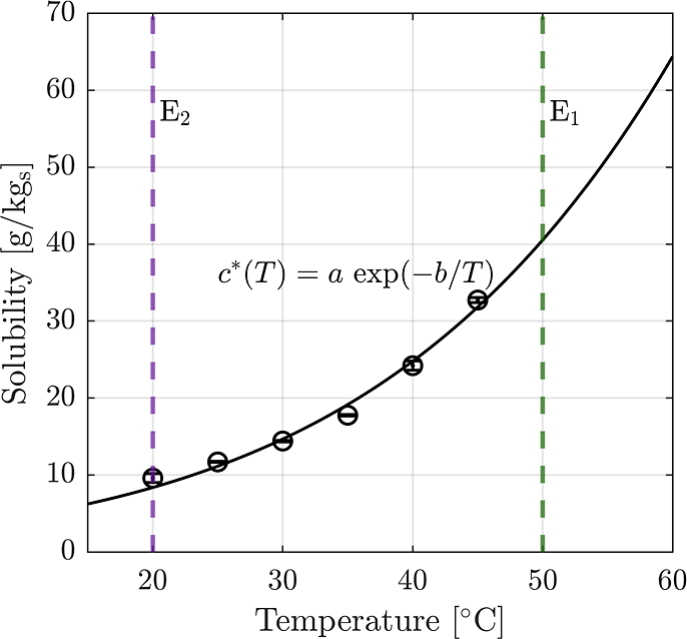
Solubility of NMPA in a mixture of 95/5 (w/w) of isopropanol (IPA) and acetonitrile (ACN). Symbols and error bars show the experimental values and the solid line represents the solubility line  fitted to the data, with  and .

To understand how much NMPA remains in solution in these two experiments, the solubility of NMPA was measured gravimetrically at various temperatures, with three repetitions for each data point. Results are shown in Figure 2.

At high temperatures, deracemization is about 4–5 times faster than that at low temperatures, yet the amount of NMPA that remains dissolved in solution at 50 °C is more than 4 times higher than that at 20 °C, namely, 40.6 g kg_s_^–1^ vs 9.6 g kg_s_^–1^. Moreover, the value of the solubility at high temperatures is on the same order of magnitude as the initial suspension density, which means that a significant amount of material is wasted if it cannot be recovered. In the next sections, we introduce and assess two processes to recover the solute from the solution at the end of a temperature cycling process performed at a high temperature level, i.e., 50 °C.

## 3 Process Variants for Enhanced Recovery

In this section, two process variants are introduced and discussed that enhance the recovery of the solute at the end of a deracemization process by temperature cycling.

### 3.1 Temperature Cycling Followed by Cooling, “TC + C”

**Figure 3:**
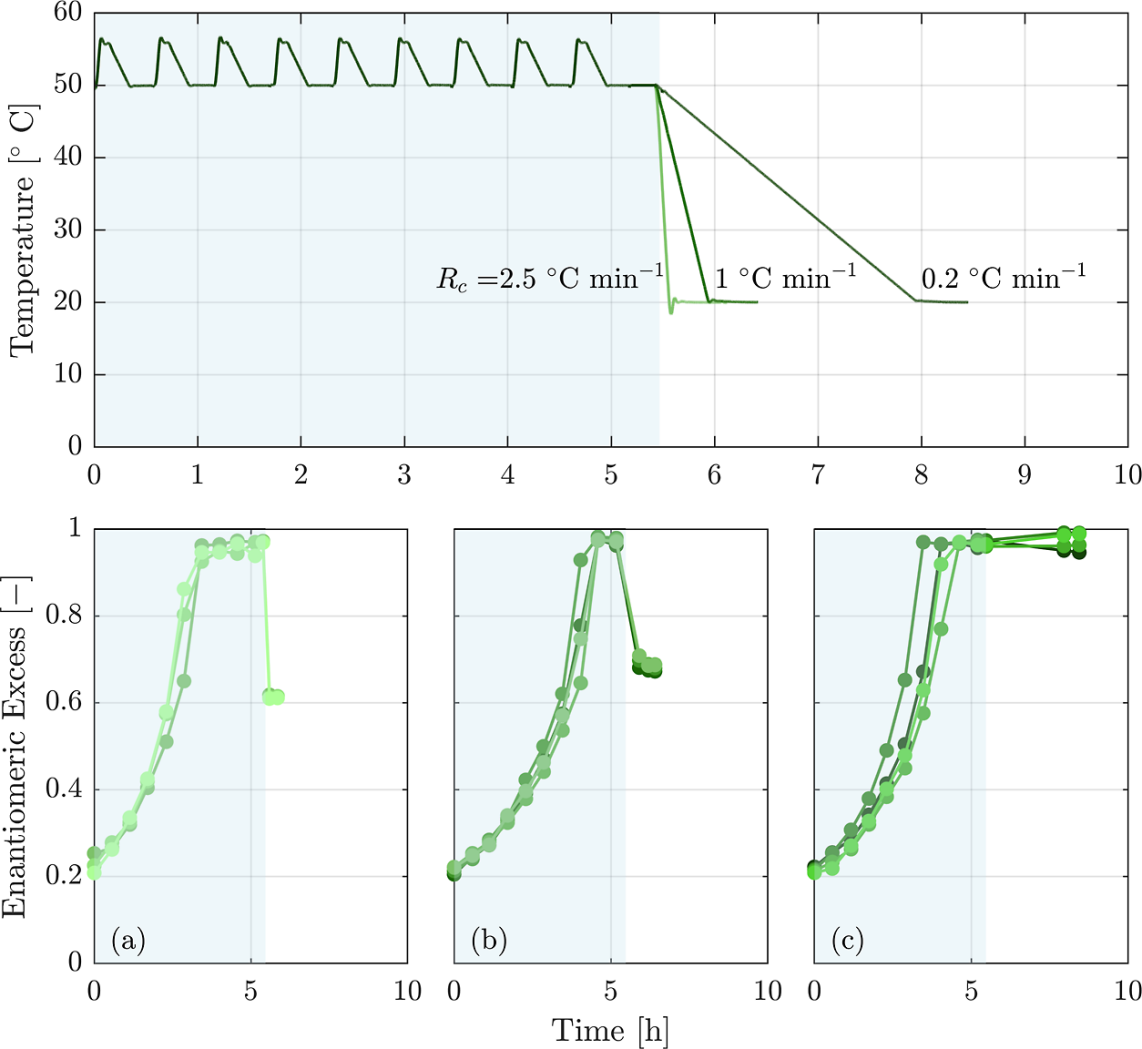
Evolution of ee for three different cooling rates. The blue-shaded area shows the temporal and purity evolution of processes during temperature cycles. The slowest rate in panel (c), 0.2 °C min^–1^, allows the recovery of solute in the form of desired crystals. In processes shown in panel (b) and (a) where cooling rates of 1 and 2.5 °C min^–1^ were implemented, product purity is lost, most likely, as a result of not only primary nucleation of undesired enantiomer but also growth and secondary nucleation of those nuclei. The worst possible case of ee loss is the condition where no material is converted (there is no catalyst or the characteristic reaction time is much larger than the rate of supersaturation buildup due to cooling). The final ee of that case is computed to 0.54 based on solubility data.

To recover the remaining solute, one may implement a linear cooling ramp at the end of the temperature cycles, i.e., once the suspended crystals reach high purity. When cooling, the solubility decreases, and more of the solute crystallizes. However, this is the case for both enantiomers, and fast cooling may lead to the nucleation of the undesired minor enantiomer, followed by the growth of its newly formed crystals, which would decrease the enantiopurity of the final product. To investigate such a process, we therefore carried out experiments at three cooling rates, under the working hypothesis that nucleation of the minority enantiomer will be favored by faster cooling: 0.2, 1, and 2.5 °C min^–1^ to cool from 50 to 20 °C (see Figure 3, top). Samples from the suspension were taken 15 and 30 min after reaching the final temperature, and ee was measured.

Figure 3, bottom, shows the resulting ee for the three experiments. While faster cooling results in a shorter overall process time, it indeed leads to a loss of ee, as seen clearly in both the 1 and 2.5 °C min^–1^ experiments.

The final ee of the experiments at 2.5 °C min^–1^ is around 0.6. For comparison, the worst-case scenario, i.e., where no material at all is converted by the chemical reaction during the cooling phase, would correspond to a final value of 0.54. This shows that at the cooling rate of 2.5 °C min^–1^ indeed only a little material reacts; only when decreasing the cooling rate significantly, there is sufficient time for the chemical reaction to convert the minor into the major enantiomer before the concentration level of the minor enantiomer becomes high enough for nucleation to take place.

The characteristic time of the chemical reaction for the system studied here has been reported earlier to be in the order of magnitudes of minutes at 20–25 °C.^40^ This information allows us to estimate the appropriate cooling rate; an unnecessarily low rate would lead to a loss in productivity, whereas too fast of a cooling, as shown here, would affect the enantiopurity of the final product.

It is worth noting that in the experiments reported here, the cooling ramp started only when ee reached a high value, i.e., 0.97 to 0.98. The energy barrier that must be overcome for primary nucleation to take place makes it possible to cool from 50 to 20 °C without loss of enantiopurity. When the ee of the crystals at the beginning of the cooling ramp was lower, the existing crystals may promote the formation of secondary nuclei, which would accelerate the dynamics of crystallization and therefore result in a more pronounced loss of enantiopurity. With respect to primary nucleation, we emphasize its volume dependence that follows from its stochastic nature; in a larger volume, the first primary nuclei will form earlier during the cooling phase, which implies that when increasing the volume, one may observe a lower final enantiopurity at the same cooling rate (see Deck and Mazzotti^46^ for a detailed discussion on primary nucleation, its stochasticity, and its interplay with secondary nucleation).

Based on the results presented in this section, we conclude that for the process variant studied here, namely, temperature cycling followed by cooling, first, the ee of the crystals at the end of the temperature cycling has to be high. Second, the racemization reaction rate has to be high so that the conversion of the undesired enantiomer into the desired one in solution is fast enough to prevent buildup of its supersaturation and its consequent nucleation.

### 3.2 Cooling Temperature Cycling, “CTC”

**Figure 4:**
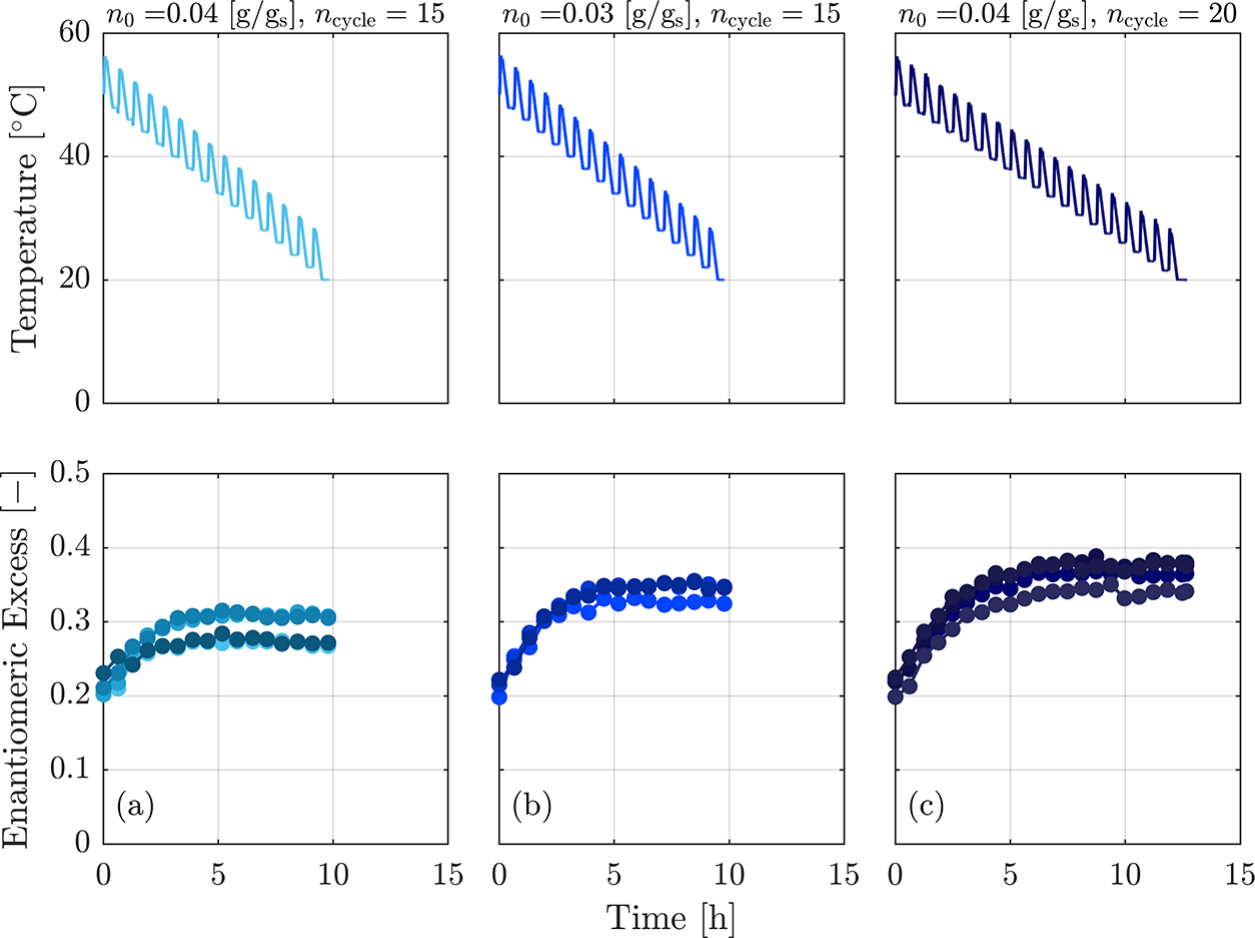
Evolution of ee in a CTC process for two initial suspension densities of *n*_0_ = 0.03 and 0.04 g g_s_^–1^ and two number of cycles of 15 and 20. As it can be seen, the experiments with smaller initial suspension density and more cycles reach a higher final value of ee, in line with expectations.

Cooling temperature cycling aims at deracemizing the suspension and at recovering the solute at the same time by merging the temperature cycles and the cooling ramp. Three examples of such a process are shown in the top row of Figure 4. Similar to a temperature cycling process, each cycle consists of four steps: a heating ramp, an isothermal step at high temperature, a cooling ramp, and an isothermal step at low temperature. However, contrary to a temperature cycling process where the initial and final temperatures of a cycle are identical, in CTC, each cycle ends at a temperature lower than the temperature at which it was started. This difference between the initial and the final temperatures of a cycle is called Δ*T*_cycle_. In addition, we introduce the term Δ*T*_process_, which denotes the difference between the initial and the final temperatures of the process, i.e., 50 and 20 °C, in the cases presented here. Obviously, Δ*T*_cycle_ can be computed when Δ*T*_process_ and the number of cycles are defined.

During the process, as the temperature gradually decreases, the kinetics of the racemization reaction and of crystal growth and dissolution slow down. Drawing insights from the experiments conducted in the preceding section, we assigned a higher number of cycles for CTC (15 and 20) compared to the cycles required for deracemization in those experiments (which were 9 cycles). We tested two suspension densities: 0.03 and 0.04 g g_s_^–1^. The heating and cooling rates, along with waiting times at high and low temperatures during the cycles, remained consistent with those used in temperature cycling. At the end of each cycle, samples were taken and the corresponding enantiomeric excess was plotted in Figure 4.

Looking at the left column, ee remains constant after a certain number of cycles. In further experiments, the suspension density was reduced to 0.03 in the experiment shown in panel (b) of the figure, and the number of cycles was set to a higher value in the experiment shown in panel (c). These changes helped to improve ee, but they were not sufficient to achieve complete enantiopurity. The results of all experiments exhibit a decrease in ee improvement as the process goes on. This behavior can be attributed to at least three factors. First, the slower kinetics at lower temperatures may lead to a smaller cycle efficiency (compare the earlier discussion on the effect of temperature on the cycle efficiency in Section 2). Second, the solubility decreases upon cooling, which leads to an increase in suspension density and to the crystallization of the minor enantiomer from the solution. Third, when maintaining a constant Δ*T*_cycle_ throughout the process, as we do here, the solubility difference of each cycle decreases as the process evolves and reaches lower temperatures, hence decreasing the maximum amount of material that can be converted.

To improve the enantiopurity in the final product, increasing the number of cycles is essential. This, however, means that the process time would be much longer than that of the TC + C alternative process variant. As the scope of this work is to investigate the best process to enable both high recovery and high productivity, the CTC with a higher number of cycles was not studied. We conclude that under the conditions explored here, TC + C outperforms CTC.

## 4 Experimental Methods

This section explains the materials and equipment as well as the experimental protocol that was used in this work.

### 4.1 Materials and Equipment

Experiments were performed with the chiral compound NMPA, synthesized in our lab following the protocol reported earlier.^47^ The compound is an imine derivative of phenylglycine that racemizes in the solution in the presence of the base 1,8-diazabicyclo[5.4.0]undec-7-ene (DBU) as catalyst.^40,47^

A mixture of 95/5 (w/w) isopropyl alcohol (IPA) and acetonitrile (ACN) was used as the solvent. The racemization of NMPA in the presence of the solvent and catalyst has been investigated elsewhere.^40^*tert*-Butyl methyl ether was used as the antisolvent to wash crystals after filtration. The racemizing agent, the solvents, and the antisolvent were purchased from Sigma-Aldrich (99% purity) and were used as received.

### 4.2 Experimental Protocol

All experiments were performed in 10 mL cylindrical glass crystallizers (2 cm diameter and 10 cm height) in an EasyMax 102 apparatus (Mettler Toledo). The device consists of two thermal blocks, each of which comprises four 10 mL crystallizers. One crystallizer per block has been equipped with a stainless steel fluorinated ethylene propylene(FEP)-coated thermocouple to monitor the temperature during the process. Polytetrafluoroethylene (PTFE) magnetic stirrers were used to stir the suspension at 1000 rpm. The stirring rate was chosen to fully suspend crystals and ensure their homogeneous distribution in the crystallizer.

The solution was equilibrated at *T*_min_ with an excess amount of suspended crystals for a minimum of 6 h. The suspension was filtered using syringe filters (PTFE, hydrophilic, 0.22 μm), and 4 g of clear solution was carefully transferred to the preheated reactors. The desired amount of DBU (6 μL g_s_^–1^) was added to the solution. Seed crystals with the desired enantiomeric excess of ee_0_ = 0.2 were prepared using the protocol described in literature^26^ and were added to the saturated solution. The crystallizers were subject to the predefined temperature profile immediately after addition of the crystals. At the beginning of the process and at the end of each temperature cycle, samples (60–80 μL, depending on the suspension density) were collected from the suspension using a precision pipet. The samples were dried by vacuum filtration using a Büchner funnel and an MS PTFE membrane filter (0.45 μm). Crystals were then washed with few droplets of antisolvent to remove residual amounts of catalyst. Few milligrams of dried crystals were transferred to HPLC vials, dissolved in acetonitrile, and analyzed via HPLC according to the protocol described in the Supporting Information (Section S2). The heating and cooling rates were fixed to 2.5 and 0.5 °C min^–1^, and the duration of the holding steps at high and low temperatures were set to 5 and 15 min, respectively.

### 4.3 Remarks on Reproducibility

Four repetitions were performed for all experiments. The evolution of ee in all experiments demonstrated excellent reproducibility. However, in a few cases (see, e.g., E_2_ in Figure 1), the ee in one of the crystallizers increased slower than that in the other three. Similarly, in panel (c) of Figure 3, the ee in one of the four crystallizers increased faster than that in the other three.

As explained previously, all four crystallizers used in each experiment are subject to identical temperature profiles in a single thermal block of EasyMax. Magnetic stirrers and crystallizing vessels were all of the same type, and for all experiments, seed crystals were taken from the same batch. To explain the variation in ee observed across repetitions of a single experiment, we highlight two potential factors.

The first reason is related to potential heterogeneities in the properties of the initial crystals. During seed batch preparation, we grind a specific mass of enantiopure NMPA with a certain mass of crystals from a racemic mixture of both enantiomers. These two fractions undergo multiple rounds of grinding following the protocol outlined in the literature.^26^ However, this protocol does not ensure a fully homogeneous distribution of crystals; i.e., it may be that repetitive sampling from the seed batch yields crystal populations of slightly different properties such as their crystal size distribution. This is in line with previous modeling work where we showed that already minor differences in the properties of the initial crystals quantitatively affect the dynamics of deracemization.^48^ A second potential reason for the experimental variability is linked to the sampling of the crystalline suspension from the crystallizer during the experiment for the measurements of the ee; carrying out such sampling in a representative manner is challenging.

## 5 Concluding Remarks

In this work, we experimentally demonstrated the effect of temperature on the deracemization of NMPA. Performing temperature cycling at high temperatures was found to be beneficial in terms of process productivity, and we rationalized this behavior using the theoretical analysis introduced earlier.^44^ The use of high temperatures, however, comes with the drawback of lower solute recovery due to the higher level of solubility. For this reason, two process variants were experimentally investigated to increase the recovery of the solute: first, temperature cycling followed by a linear cooling ramp (TC + C), and second, the integration of temperature cycles into cooling crystallization, termed CTC.

In a comprehensive experimental campaign, we demonstrated that faster deracemization is achieved in the former process (TC + C), which is also easier to design and implement than the latter. However, the choice of an appropriate cooling rate is essential to avoid nucleation of the undesired crystals. In contrast, in a CTC process, several factors decrease the rate of deracemization, which make the process less productive and its design much more challenging; thus, such a cooling temperature cycling process is not of interest for further investigation.

## Supporting Information Available

This document contains detailed information on the HPLC device and instrument protocol that was used for enantiomeric excess measurement of samples taken from the suspension, tests on the thermal stability of NMPA, and additional experiments we have performed to support the experiments reported in the main body (PDF)
