# Supplementary material for: On Solute Recovery and Productivity in Chiral Resolution through Solid-State Deracemization by Temperature Cycling
Source: Cryst Growth Des. 2024 Apr 23;24(9):3925–32. doi: 10.1021/acs.cgd.4c00233 (PMC11066838; doi:10.1021/acs.cgd.4c00233)
Supplement: Supplementary file 1 — cg4c00233_si_001.pdf [file cg4c00233_si_001.pdf]

Supporting information for: On solute recovery and productivity in chiral resolution through solid-state deracemization by temperature-cycling

Mercedeh Sadat Hosseinalipour, Leif-Thore Deck, and Marco Mazzotti*

Institute of Energy and Process Engineering, ETH Zurich, 8092 Zurich, Switzerland

E-mail: marco.mazzotti@ipe.mavt.ethz.ch

Phone: +41 44 632 24 56. Fax: +41 44 632 11 41

Abstract

This document contains supporting information that provide additional details of the experimental work carried out in the main body. The following materials are supplied. In Section S.1 we provide detailed information on the HPLC device and instrument protocol that were used for enantiomeric excess measurement of samples taken from the suspension. We report in Section S.2 tests on the thermal stability of NMPA. And finally in Section S.3 we report additional experiments we have performed to support the experiments in the main body.

S Supporting Information

S.1 HPLC Protocol for Sample Analysis

To monitor the evolution of the enantiomeric excess during the process, a sample containing 60-100 µL of suspension was vacuum filtered and washed with anti-solvent at the end of each temperature cycle. In addition to these samples, a zero-th sample was taken from the suspension before the temperature cycles were started to determine the initial enantiomeric excess (ee_0). The samples were transferred to crimp top clear glass 1.5 mL HPLC vials and dissolved in acetonitrile for measurements. All measurements were carried out in a HPLC apparatus (DIONEX UltiMate 3000 series) that was equipped with a quaternary pump and DAD detector (Thermo Scientific, Reinach, Switzerland). Measurements were carried out at UV-VIS 213 nm on a CHIRALPAKL AY 20 μm stationary phase packed in a 250 mm $\times$ 4.6 mm column to separate enantiomers. From each HPLC vial, 2 μL was sampled and injected to the column kept at 27 °C. Pure acetonitrile was used as the mobile phase with a flow rate of 2 mL min^{-1}. Each measurement lasted 6 minutes and the retention times were found to be 2.07 min for (L)-NMPA and 3.26 min for (D)-NMPA as shown in Figure S1. The enantiomeric excess was calculated using the ratio of the HPLC peak areas for both enantiomers.

S.2 Stability of NMPA

Since deracemization experiments for NMPA have not been reported in the literature at temperatures of 50 ° and above, we have analyzed products of some of the experiments by ^{1}H-NMR to verify the thermal stability of the compound. The ensuing spectra were compared with values reported in the supplementary information of the publication by Iggland et al.[1], whereby we observed a quantitative agreement. This indicates that no degradation of NMPA took place under the given experimental conditions. This is further confirmed by the fact that the peaks measured using HPLC in all experiments appeared at the same retention times and exhibited regular shapes.

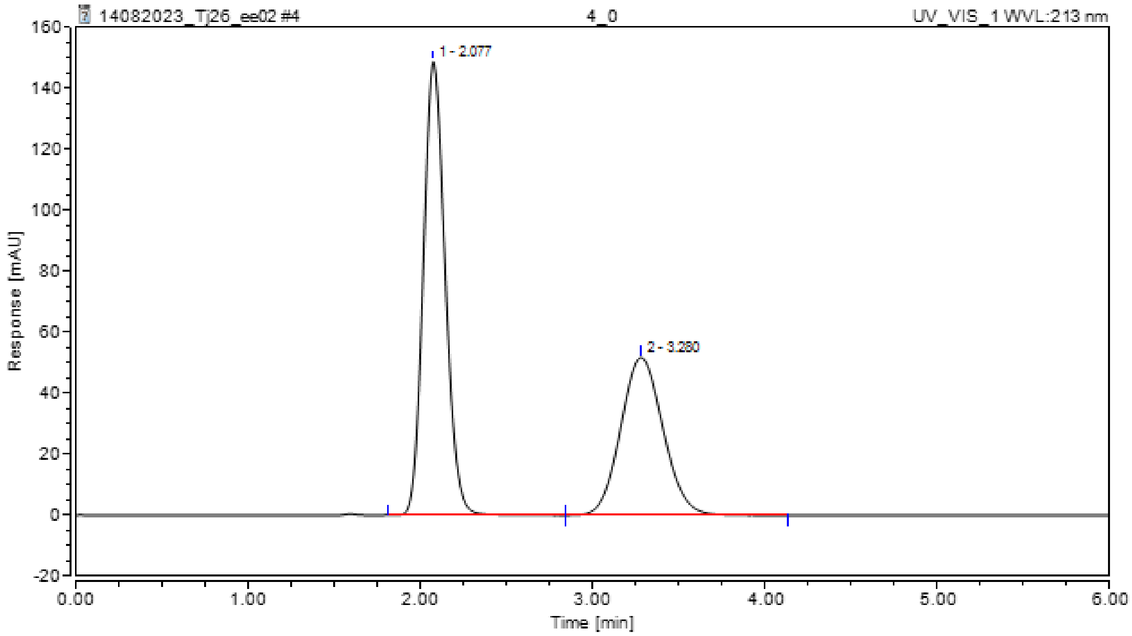

Figure S1: Example of a chromatogram of a sample analysed by above-mentioned HPLC protocol.

S.3 Additional Experiment to Improve Enantiomeric Excess

The enantiomeric excess in temperature cycling experiments reported both in this work, as well as in the literature,[2–4] in most cases does not reach a final value of $ee = 1$, but approaches a plateau at a slightly lower value on the order of 0.97–0.98. To understand the underlying reason behind this observation, we ran an experiment during which we decreased the cycle amplitude over time. We conjectured that nucleation of the undesired enantiomer during the cooling step of a temperature cycle may affect the attainable enantiomeric excess. By decreasing the cycle amplitude, the difference in supersaturation between T_{max} and T_{min} decreases and thus, nucleation is expected to become less relevant. The results reported in Figure S2 show that decreasing the difference between the high and low temperature of the cycles does not lead to complete enantiopurity, which indicates that no relevant nucleation of the undesired enantiomer takes place, in contrast with our hypothesis. The reason behind this observation may thus instead be related to not yet understood shortcomings in the measurement methodology.

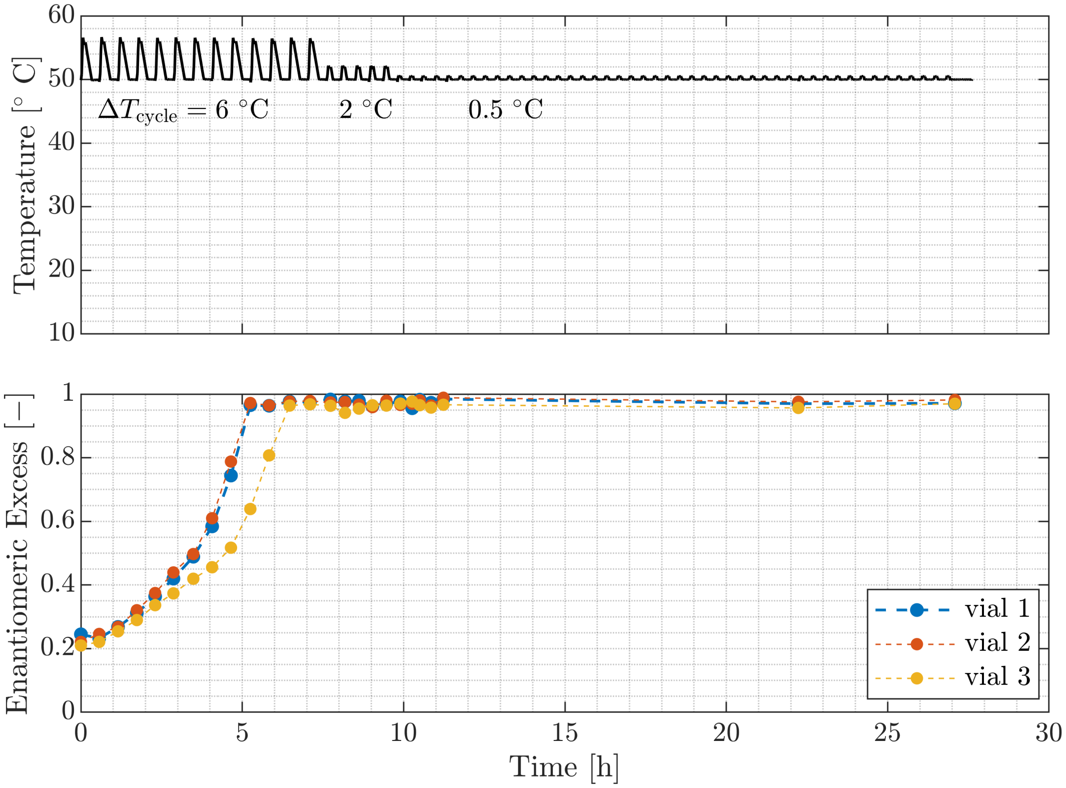

Figure S2: Experiment with decreasing cycle amplitude aimed at improving the final enantiomeric excess.

References

(1) Iggland, M.; Fernández-Ronco, M. P.; Senn, R.; Kluge, J.; Mazzotti, M. Complete solid state deracemization by High Pressure Homogenization. *Chemical Engineering Science* **2014**, *111*, 106–111.

(2) Breveglieri, F.; Maggioni, G. M.; Mazzotti, M. Deracemization of NMPA via temperature cycles. *Crystal Growth & Design* **2018**, *18*, 1873–1881.

(3) Breveglieri, F.; Baglai, I.; Leeman, M.; Noorduin, W. L.; Kellogg, R. M.; Mazzotti, M. Performance analysis and model-free design of deracemization via temperature cycles. *Organic Process Research & Development* **2020**, *24*, 1515–1522.

(4) Breveglieri, F.; Bodák, B.; Mazzotti, M. Deracemization via periodic and non-periodic tem-

perature cycles: rationalization and experimental validation of a simplified process design approach. *Organic Process Research & Development* **2021**, *25*, 2551–2565.
